# Prognostic outcomes of cytomegalovirus reactivation after autologous stem cell transplantation

**DOI:** 10.7150/ijms.79285

**Published:** 2023-01-09

**Authors:** Byung-Hyun Lee, Min Ji Jeon, Eun Sang Yu, Ka-Won Kang, Dae Sik Kim, Se Ryeon Lee, Yong Park, Hwa Jung Sung, Chul Won Choi, Byung Soo Kim

**Affiliations:** Department of Internal Medicine, Korea University College of Medicine, Seoul, Korea.

**Keywords:** cytomegalovirus, autologous stem cell transplantation, multiple myeloma, prognosis

## Abstract

**Background:** Cytomegalovirus (CMV) reactivation is a common complication in patients undergoing allogeneic stem cell transplantation. However, the incidence of CMV reactivation is low after autologous stem cell transplantation (auto-SCT), and the prognostic value of CMV reactivation remains controversial. Moreover, reports on late CMV reactivation after auto-SCT are limited. We aimed to analyze the association between CMV reactivation and survival outcomes and develop a predictive model for late CMV reactivation in patients undergoing auto-SCT.

**Methods:** Data of 201 patients who underwent SCT at the Korea University Medical Center from 2007 to 2018 were collected. We analyzed prognostic factors for survival outcomes after auto-SCT and risk factors for late CMV reactivation using a receiver operating characteristic curve. Then, we developed a predictive risk model for late CMV reactivation based on results of the risk factor analysis.

**Results:** Early CMV reactivation was significantly associated with better overall survival (OS) (hazard ratio [HR], 0.329; *P* = 0.045) in patients with multiple myeloma; however, no significant differences were observed in patients with lymphoma. For late CMV reactivation, a serum lactate dehydrogenase level greater than the upper limit of normal (HR, 2.251; *P* = 0.027) and late CMV reactivation (HR, 2.964; *P* = 0.047) were independent risk factors for poor OS, while lymphoma diagnosis (vs. multiple myeloma; HR, 0.389; *P* = 0.016) was an independent risk factor for good OS. In risk factor analysis for late CMV reactivation, T-cell lymphoma diagnosis (odds ratio [OR], 8.499; *P* = 0.029), ≥ two prior chemotherapies (OR, 8.995; *P* = 0.027), failure to achieve complete response (CR) after transplantation (OR, 7.124; *P* = 0.031), and early CMV reactivation (OR, 12.853; *P* = 0.007) were significantly associated with late CMV reactivation. To develop the predictive risk model for late CMV reactivation, a score (1 to 1.5) was assigned for each of the above-mentioned variables. The optimal cutoff value (1.75 points) was calculated using the receiver operating characteristic curve. The predictive risk model showed good discrimination, with an area under the curve of 0.872 (standard error, 0.062; *P* < 0.001).

**Conclusions:** Late CMV reactivation was an independent risk factor for inferior OS, whereas early CMV reactivation was associated with better survival in patients with multiple myeloma. This risk prediction model could be helpful in identifying high-risk patients who require monitoring for late CMV reactivation and potentially benefit from prophylactic or preemptive therapy.

## Introduction

Cytomegalovirus (CMV) reactivation is a frequent complication in patients undergoing stem cell transplantation (SCT). It is commonly known to increase transplant-related mortality and lead to the development of specific disease after SCT, including hepatitis, pneumonia, gastroenteritis, and retinitis 1. In contrast, some previous studies have reported that CMV reactivation after allogeneic SCT (allo-SCT) is associated with a reduced risk of early relapse in patients with acute myeloid leukemia (AML); however, it was not related to relapse in other diseases, such as chronic myeloid leukemia (CML), acute lymphoblastic leukemia, or lymphoma 2. This finding is contradictory to previous findings on CMV reactivation after SCT 3. Studies have reported that early CMV reactivation was associated with decreased relapse rate in patients with CML 4 and lymphoma 5; however, another study showed that CMV reactivation was associated with increased non-relapse mortality and lower overall survival (OS) in patients with AML, CML, and myelodysplastic syndrome (MDS) 6. In a study by Thomson et al., no association was found between CMV reactivation and relapse risk in patients with AML who received a novel immunosuppressive agent, alemtuzumab 7. In contrast, asymptomatic CMV reactivation was found to be a prognostic factor for better OS in a small retrospective study conducted in allo-SCT recipients 8.

However, CMV reactivation is infrequently observed after autologous SCT (auto-SCT). The incidence of CMV reactivation after auto-SCT is considerably lower than that after allo-SCT (4-9% vs. 21-38%) 9. Moreover, symptomatic CMV reactivation or infection is rare in the auto-SCT setting 10. However, the incidence of CMV reactivation is high in patients with lymphoma and multiple myeloma based on treatment and disease-associated characteristics 10, 11. Although CMV was found to cause life-threatening complications, such as pneumonia, in patients who underwent auto-SCT 12, most studies were conducted before the introduction of novel chemotherapeutic drugs 10. Thus, currently available information is limited to the clinical progression and implications of CMV reactivation after auto-SCT. Although immune reconstitution after auto-SCT could affect the clinical outcomes of transplant recipients 13, the effect of CMV reactivation on prognosis after auto-SCT remains controversial.

This study aimed to evaluate the association between CMV reactivation and survival outcomes in patients undergoing auto-SCT. Based on the results of this study, we developed a predictive model for late CMV reactivation following auto-SCT. This model could help identify patients who may potentially benefit from optimized strategies for monitoring, preventing, and treating late CMV reactivation after auto-SCT.

## Methods

### Patients

The Korea University bone marrow transplantation registry is a longitudinal cohort containing data of patients who underwent SCT at the Korea University Medical Center (Korea University Anam, Guro, and Ansan Hospitals) from January 2007 to December 2018. According to this registry, 201 patients underwent auto-SCT: 107 with multiple myeloma; 50 with B-cell lymphoma; 35 with T-cell lymphoma; 8 with Hodgkin lymphoma, and 1 with amyloid light-chain (AL) amyloidosis. Patients with AL amyloidosis were excluded because critical medical data were missing. Of the 200 patients, 50 were not tested for CMV reactivation. Thus, we retrospectively analyzed data from 150 patients with multiple myeloma (n = 80), B-cell lymphoma (n = 39), T-cell lymphoma (n = 26), and Hodgkin lymphoma (n = 5). All methods were performed in accordance with relevant guidelines and regulations. This study was approved by the Institutional Review Board of the Korea University Medical Center, with a waiver of informed consent for the collection and analysis of retrospective data.

### Definitions

CMV reactivation was defined as the detection of CMV viremia based on viral load greater than the lower limit of detection (LOD) using quantitative polymerase chain reaction (qPCR). Early and late CMV reactivations were defined as CMV reactivation 100 days before and after transplantation, respectively. The diagnosis of CMV disease requires identification of CMV in biopsy specimens. Treatment responses were assessed according to the International Myeloma Working Group response criteria 14 for multiple myeloma and the Lugano classification 15 for lymphoma. OS was measured from the start of transplantation until death from any cause. Progression-free survival (PFS) was defined as the time from the start of transplantation to disease progression or death from any cause. High-risk cytogenetics in multiple myeloma was defined as t(4;14), t(14;16), del(17/17p), *TP53* deletion, or chromosome 1 abnormalities, including gain(1q) and del(1p).

### CMV monitoring and management

CMV reactivation was monitored once or twice a week by qPCR in all patients until day 100 after transplantation and thereafter, only if clinically indicated. All patients received acyclovir for antiviral prophylaxis. CMV reactivation was preemptively treated with ganciclovir according to the physicians' decisions.

### Prediction model

A predictive model for late CMV reactivation was constructed based on the results of risk factor analysis using a stepwise logistic regression method. The receiver operating characteristic (ROC) curves and area under the curve (AUC) were used to evaluate the performance and prediction accuracy of the model. The optimal cutoff point for estimating late CMV reactivation was identified as the point at which the AUC value was maximal.

### Statistical analysis

Categorical variables were evaluated using the chi-square test or Fisher's exact test, and continuous variables were evaluated using the Student's t-test. Backward stepwise logistic regression analysis was used to estimate the association between late CMV reactivation and clinical variables. Survival curves were estimated using the Kaplan-Meier method and compared using the log-rank test. Cox proportional hazards model with the backward stepwise elimination method was used to analyze the association between survival rates and other prognostic variables. All tests were two sided, and *P*-values < 0.05 were considered significant. Statistical analyses were performed using Statistical Package for the Social Sciences version 25.0 software (IBM Corporation, New York, NY, USA) and GraphPad Prism version 9.0.1 software (GraphPad Software Inc., San Diego, CA, USA).

## Results

### Patient characteristics

The baseline characteristics are summarized in Table 1. The median age of patients who underwent auto-SCT was 52 (17-69) years in all patients, 53 (27-69) years in patients with multiple myeloma, 51 (21-64) years in patients with B-cell lymphoma, 51 (17-65) years in patients with T-cell lymphoma, and 29 (19-54) years in patients with Hodgkin lymphoma. Of the 200 patients, 150 (75.0%) were tested for CMV reactivation. CMV reactivation was observed in 57 (38.0%) patients based on the LOD and in 28 (18.6%) patients based on the limit of quantification (LOQ). The median duration of CMV reactivation was 14 days (3-346). Preemptive therapy was provided to 24 patients, all of whom received ganciclovir. CMV reactivation was observed in 57 (38.0%) patients before 100 days and 7 (4.7%) patients after 100 days. Sixty-five (32.5%) patients received two or more chemotherapy lines before transplantation, and all patients underwent single transplantation.

### Survival analyses in relation to CMV reactivation

**Figure 1:**
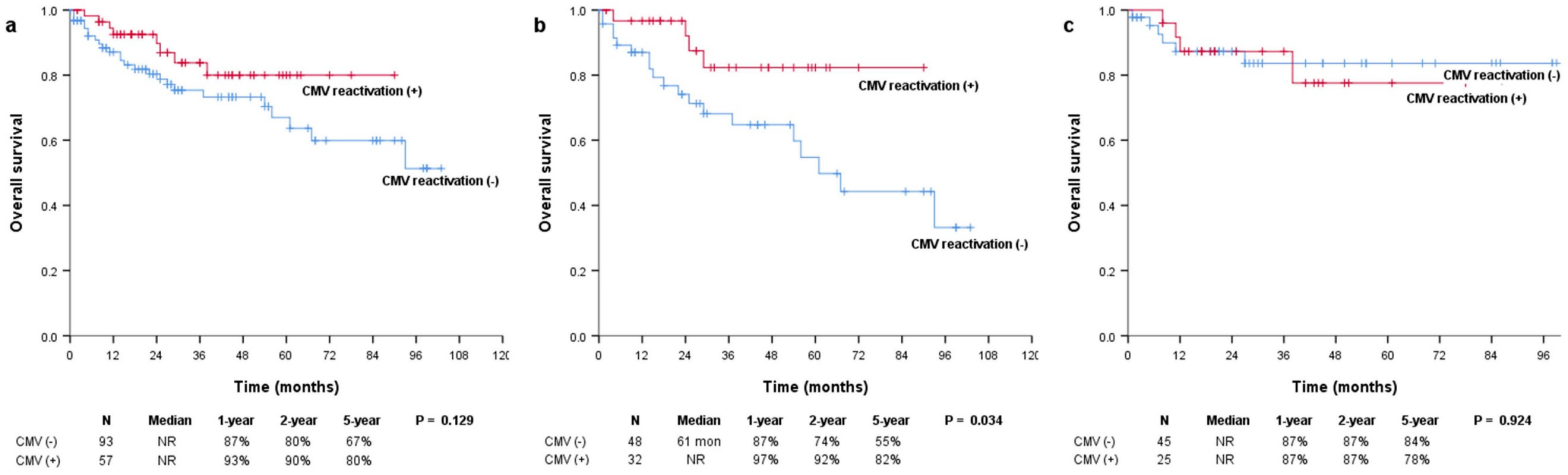
Kaplan-Meier survival curves for OS in (a) all patients and patients with (b) multiple myeloma and (c) lymphoma. OS: overall survival.

**Figure 2:**
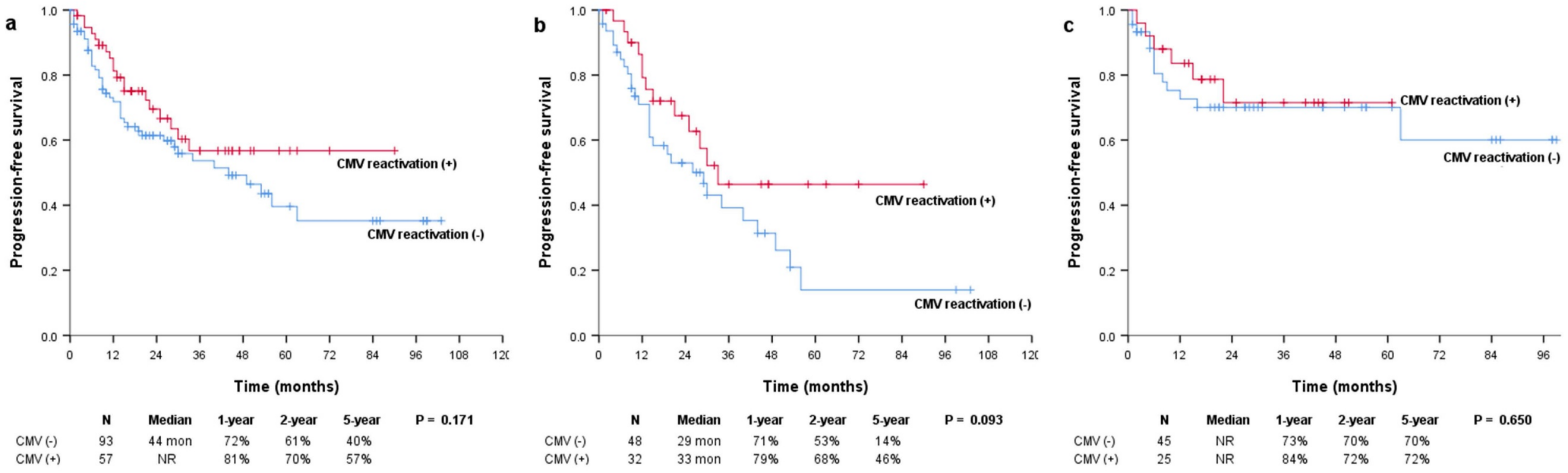
Kaplan-Meier survival curves for PFS in (a) all patients and patients with (b) multiple myeloma and (c) lymphoma. PFS: progression-free survival.

The median OS was not reached in either the positive or negative CMV reactivation group, and there were no significant differences between them (*P* = 0.129; Figure 1a). However, in the subgroup of patients with multiple myeloma, OS was significantly more prolonged in patients with CMV reactivation than in those without CMV reactivation (median, not reached vs. 61 months; *P* = 0.034) (Figure 1b). In patients with and without CMV reactivation, the 1-year OS rates were 97% and 87%, the 2-year OS rates were 92% and 74%, and the 5-year OS rates were 82% and 55%, respectively. There were no significant differences in the OS of patients with lymphoma (median, not reached vs. not reached; *P* = 0.924) (Figure 1c). The median PFS was not reached and was 44 months for patients with and without CMV reactivation, respectively (*P* = 0.171; Figure 2a). In patients with multiple myeloma, patients with CMV reactivation showed better PFS (median, 33 months vs. 29 months; *P* = 0.093) (Figure 2b) than patients without CMV reactivation; however, these results did not reach statistical significance. There were no significant differences in PFS between patients with lymphoma (median, not reached vs. not reached; *P* = 0.650) (Figure 2c).

Then, to confirm the association between CMV reactivation and survival prognosis of multiple myeloma, we conducted univariable and multivariable analyses including other relevant clinical factors. The characteristics of patients with multiple myeloma are summarized in Table 2, and the results are shown in Table 3. In the univariable analyses, CMV reactivation (hazard ratio [HR], 0.329; *P* = 0.045) was a significant favorable prognostic factor, whereas a serum lactate dehydrogenase (LDH) level higher than the upper limit of normal (HR, 2.894; *P* = 0.012) and high-risk cytogenetics (HR, 4.213; *P* = 0.002) were significant unfavorable prognostic factors. The high revised international staging system (R-ISS) stage was associated with poor survival, with borderline significance (HR, 2.459; *P* = 0.080). In the multivariable analysis using the backward stepwise elimination method, including variables of *P* < 0.2 in the univariable analyses, hemoglobin (Hb) level, platelet (PLT) count, type of M-protein, LDH level higher than the upper limit of normal, high-risk cytogenetics, R-ISS stage III, and CMV reactivation (HR, 0.213; 95% confidence interval [CI], 0.062-0.739; *P* = 0.015) were significant favorable prognostic factors, whereas a serum LDH level higher than the upper limit of normal (HR, 2.483; 95% CI, 1.046-5.893; *P* = 0.039) and non-IgG type of M-protein (HR, 2.614; 95% CI, 1.037-6.587; *P* = 0.042) were significant unfavorable prognostic factors. High-risk cytogenetics was associated with poor survival with borderline significance (HR, 2.404; 95% CI, 0.936-6.178; *P* = 0.068). Other factors did not show a significant association with OS in patients with multiple myeloma.

Furthermore, to analyze the effect of late CMV reactivation on OS, we also conducted Cox regression analyses including relevant prognostic factors (Table 4). In the univariable analyses, no factor was significantly associated with OS. In the multivariable analysis using the backward stepwise elimination method, including variables with *P* < 0.2 in the univariable analyses, Hb level, LDH level higher than the upper limit of normal, diagnosis of lymphoma, failure to achieve complete response (CR), two or more previous chemotherapy lines, and late CMV reactivation were significantly associated with OS. A poor prognosis was significantly associated with a serum LDH level higher than the upper limit of normal (HR, 2.251; 95% CI, 1.095-4.627; *P* = 0.027) and late CMV reactivation (HR, 2.964; 95% CI, 1.014-8.667; *P* = 0.047), whereas a good prognosis was significantly associated with the diagnosis of lymphoma (vs. diagnosis of multiple myeloma; HR, 0.389; 95% CI, 0.181-0.837; *P* = 0.016). In summary, in contrast to CMV reactivation, late CMV reactivation was a prognostic factor of poor survival in patients undergoing auto-SCT. Based on these results, we developed a new scale to predict late CMV reactivation.

### Prediction model for late CMV reactivation

To investigate the predictors of late CMV reactivation, we conducted a multivariable logistic regression analysis using the backward stepwise elimination method and included all clinically relevant variables. The results are shown in Table 5. Late CMV reactivation was independently predicted by a diagnosis of T-cell lymphoma (odds ratio [OR], 8.499; 95% CI, 1.242-58.151; *P* = 0.029), two or more previous chemotherapy lines (OR, 8.995; 95% CI, 1.280-63.216; *P* = 0.027), failure to achieve CR after SCT (OR, 7.124; 95% CI, 1.202-42.210; *P* = 0.031), and early CMV reactivation (OR, 12.853; 95% CI, 1.990-82.996; *P* = 0.007).

**Figure 3:**
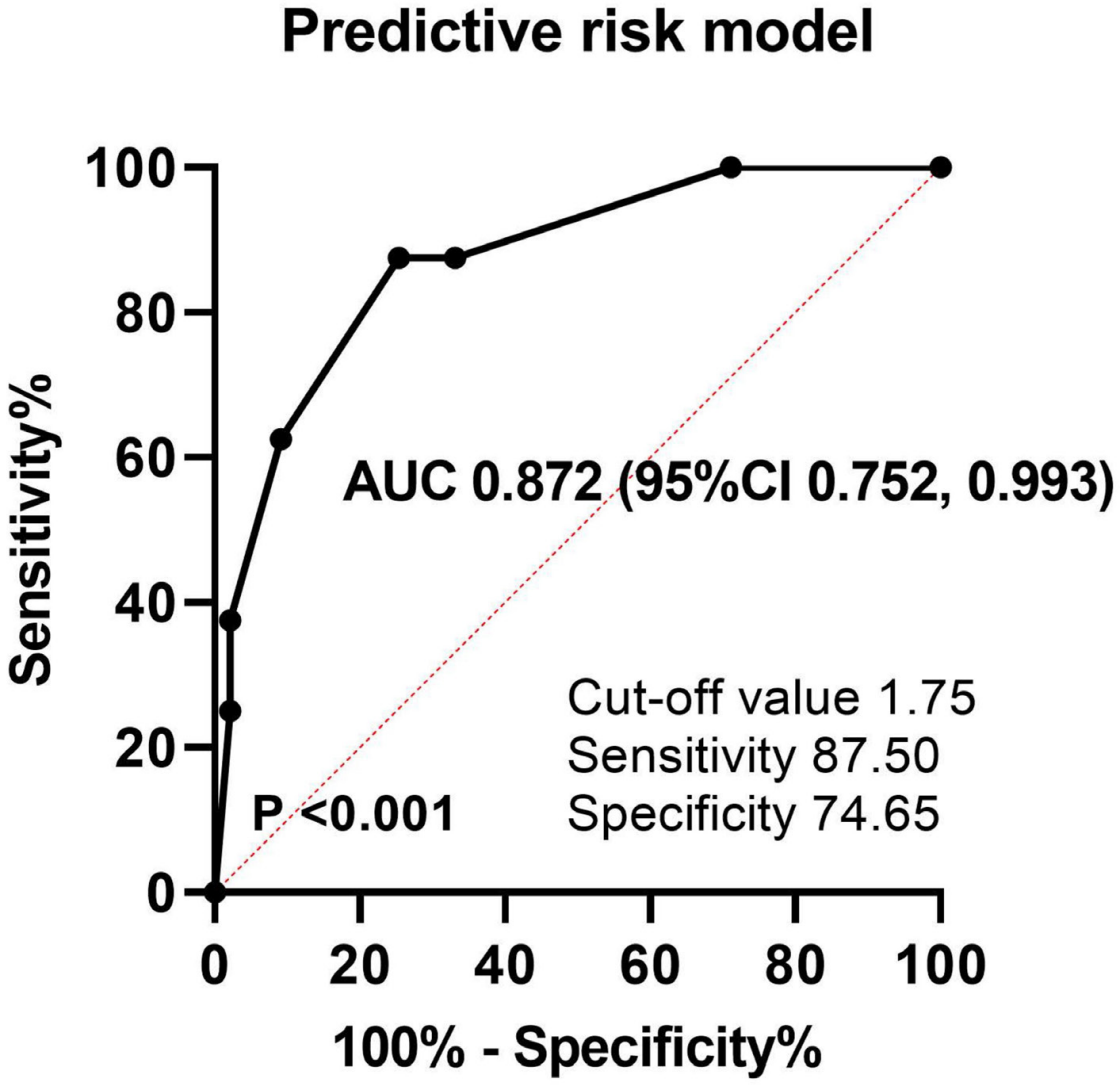
ROC curve and AUC analysis for late CMV reactivation. AUC: area under the curve, ROC: receiver operating characteristic.

Then, to develop the predictive risk model for late CMV reactivation, a score from 1 to 1.5 was assigned for each of the four variables based on estimated coefficients in logistic analysis: diagnosis of T-cell lymphoma (1 point), two or more previous chemotherapy lines (1 point), failure to achieve CR (1 point), and early CMV reactivation (1.5 points). The total scores were calculated by adding these four values (0-4.5). The optimal cutoff value for the estimated late CMV reactivation was 1.75 points calculated using the ROC curve, with a sensitivity of 87.50% and specificity of 74.65% (Figure 3). Thus, 43 patients (28.7%) were assigned to the low-risk group (score < 1.75) and 107 patients (71.3%) to the high-risk group (score ≥ 1.75). The predictive risk model showed good discrimination ability with an AUC of 0.872 (standard error, 0.062; 95% CI, 0.752-0.993; *P* < 0.001).

## Discussion

In our retrospective cohort analysis, late CMV reactivation was associated with poor survival in auto-SCT recipients, whereas early CMV reactivation was associated with better survival in patients with multiple myeloma. Based on these results, we constructed a statistical model using relevant clinical parameters to predict late CMV reactivation in patients who underwent auto-SCT. Our model accurately predicted late CMV reactivation and identified patients who required monitoring for late CMV reactivation and who potentially benefit from prophylactic or preemptive therapy.

The prognostic value of CMV reactivation after SCT has been evaluated in several previous studies; however, these studies have shown contradictory results. A single-center study showed that CMV reactivation 100 days before allo-SCT was significantly associated with a 32% decrease in the risk of relapse in patients with AML 2. In another study of patients with AML, CMV reactivation was an independent protective factor for the risk of relapse (HR, 0.77; *P* = 0.04) 16. Furthermore, CMV reactivation has been associated with a lower risk of relapse in patients with CML and lymphoma 4, 5. In contrast, CMV reactivation was an independent risk factor for non-relapse mortality after allo-SCT in another study, which reported a 31% overall increase in the risk of death without relapse 2. A study using data from a large multicenter research database concluded that CMV is significantly associated with higher non-relapse mortality rate and lower OS in patients with AML, CML, and MDS 6. In a study of patients with aplastic anemia who underwent allo-SCT, CMV reactivation was a significant independent prognostic factor for shorter OS in a multivariable analysis (HR, 1.65; *P* = 0.42) 17. Thus, although it remains controversial, CMV reactivation seems to be an adverse prognostic factor in the allo-SCT setting.

CMV reactivation is a rare complication (2.9%) in patients who undergo auto-SCT 10, and previous reports investigating CMV reactivation or infection in relation to auto-SCT are limited. The major risk factors for CMV reactivation are well-known CMV serostatus, acute or chronic graft-versus-host disease (GVHD), donor type (unrelated or mismatched donor), and immunosuppressive treatment 18, 19. However, these factors are not applicable in the auto-SCT setting. In a retrospective study that included 324 patients who underwent auto-SCT, the risk of CMV reactivation was positively associated with the diagnosis of non-Hodgkin lymphoma (OR, 4.9; *P* = 0.01) or multiple myeloma (OR, 4.6; *P* = 0.03), progressive disease at transplantation (OR, 4.9; *P* = 0.03), and age (OR, 1.04; *P* = 0.01) 11. Tandem transplantation was also a significant risk factor for CMV reactivation (OR, 5.112; *P* = 0.02) in patients with multiple myeloma 20. A retrospective study reported that early CMV reactivation, younger patient age, and acute GVHD were significant risk factors for late CMV reactivation after allo-SCT 21. However, the risk factors associated with late CMV reactivation in patients who underwent auto-SCT have not been extensively studied.

The present study analyzed the risk factors for late CMV reactivation after auto-SCT because late CMV reactivation was an independent predictor of poor survival after auto-SCT in our setting. According to a previous report, OS was significantly lower in patients with lymphoma with CMV reactivation than in those without CMV reactivation; however, no difference in OS was observed in patients with multiple myeloma 11. These findings were partially comparable to our results that CMV reactivation was associated with favorable OS in patients with multiple myeloma but not in patients with lymphoma. Nevertheless, the prognostic implications of CMV reactivation in relation to auto-SCT remain unclear. Among 7 patients with late CMV reactivation, 2 (28.6%) achieve CR at SCT and 5 (71.4%) received two or more line of therapy before SCT. Among 57 patients with early CMV reactivation, 35 (61.4%) achieve CR at SCT and 13 (22.8%) received two or more line of therapy before SCT. Based on our data, patients with late CMV reactivation have more advanced and refractory disease than those with early CMV reactivation and this could affect different survival outcomes between early and late CMV reactivation. We also found that a diagnosis of T-cell lymphoma, two or more chemotherapy lines before transplantation, failure to achieve CR, and early CMV reactivation were significant risk factors for late CMV reactivation. Early CMV reactivation was significantly associated with late CMV reactivation based on our data on auto-SCT, similar to that in a previous study 21; however, patient age was not associated with late CMV reactivation in the current study. We could not consider all relevant factors for late CMV reactivation in this analysis, and further evaluation is required to confirm our results.

The present study has several limitations. First, it was conducted based on a retrospective analysis of a relatively small cohort. Thus, confirmatory conclusions could not be drawn from the results of this study. Second, we used data obtained from multicenter and longitudinal cohorts; thus, the testing methods, including sensitivity (LOD) and detection range (LOQ) for CMV DNA viral load by qPCR, were not consistent. Third, CMV reactivation and prognostic values could be affected by specific chemotherapeutic agents, including immunologic or cancer-specific treatment. Future studies should consider the effects of novel agents before and after transplantation. Fourth, we could not perform immunological or functional analyses. The absence of CMV-specific immunity by 3 months after SCT was significantly associated with late CMV reactivation and increased mortality 22. These experimental analyses would help clarify the discrepancy between early and late CMV reactivation in relation to prognosis after auto-SCT. Finally, although our risk prediction model showed good performance based on the AUC value (0.872), we could not conduct a validation analysis due to the low incidence of late CMV reactivation (4.7%) and absence of available external datasets. Additional analyses are needed to validate our model and determine whether it can be used to effectively predict late CMV reactivation.

In conclusion, late CMV reactivation is a rare complication in patients undergoing auto-PBSCT; however, it is also associated with poor survival outcomes. In addition, we found that early CMV reactivation was an independent risk factor for favorable survival in patients with multiple myeloma receiving auto-SCT. Prediction of late CMV reactivation using our model could be helpful in identifying high-risk patients who require monitoring for late CMV reactivation and might provide personalized CMV treatment strategies to clinicians.

## Figures and Tables

**Table 1:** Baseline characteristics of the study cohort

	Total, n (%) (n = 200)	MM, n (%) (n = 107)	B-lymphoma, n (%) (n = 50)	T-lymphoma, n (%) (n = 35)	HL, n (%) (n = 8)
**Age, median years (range)**	52 (17-69)	53 (27-69)	51 (21-64)	51 (17-65)	29 (19-54)
**Sex**					
Male	125 (62.5)	66 (61.7)	32 (64.0)	21 (60.0)	6 (75.0)
Female	75 (37.5)	41 (38.3)	18 (36.0)	14 (40.0)	2 (25.0)
Patients tested for CMV reactivation	150 (75.0)	80 (74.8)	39 (78.0)	26 (74.3)	5 (62.5)
**CMV RT-PCR, copies**					
< LOD	93 (62.0)	48 (60.0)	28 (71.8)	13 (50.0)	4 (80.0)
LOD-LOQ	29 (19.3)	16 (20.0)	6 (15.4)	7 (26.9)	0
LOQ-10000	20 (13.3)	12 (15.0)	4 (10.3)	4 (15.4)	0
≥ 10000	8 (5.3)	4 (5.0)	1 (2.5)	2 (7.7)	1 (20.0)
CMV disease (pathology confirmed)	1 (0.7)	1 (1.3)	0	0	0
**CMV treatment**					
Ganciclovir	24 (100)	12 (100)	6 (100)	5 (100)	1 (100)
Others	0	0	0	0	0
CMV duration, median days (range)	14 (3-346)	14 (3-57)	11 (3-45)	15 (7-346)	37 (37)
**Timing of CMV reactivation (≥ LOD)**					
Early (before day 100)	57 (38.0)	32 (40.0)	11 (28.2)	13 (50.0)	1 (20.0)
Late (after day 100)	7 (4.7)	1 (1.3)	2 (5.1)	3 (11.5)	1 (20.0)
Both	5 (3.3)	0	2 (5.1)	2 (7.7)	1 (20.0)
**Lines of therapy before transplantation**					
1	135 (67.5)	81 (75.7)	29 (58.0)	25 (71.4)	0
≥ 2	65 (32.5)	26 (24.3)	21 (42.0)	10 (28.6)	8 (100)
**Number of transplantations**					
1	200 (100)	107 (100)	50 (100)	35 (100)	8 (100)
≥ 2	0	0	0	0	0

Note: Data of one patient with amyloidosis are not shown. CMV: cytomegalovirus, MM: multiple myeloma, HL: Hodgkin lymphoma, LOD: limit of detection, LOQ: limit of quantification.

**Table 2:** Clinical characteristics according to early CMV reactivation in patients with multiple myeloma

Parameter, n (%) / median (range)	Total (n = 80)	CMV reactivation + (≥ LOD), (n = 32)	CMV reactivation - (< LOD), (n = 48)	*P*
Age	54 (27-69)	57 (30-69)	51 (27-65)	0.317
**Sex**				
Male	48 (60.0)	15 (46.9)	33 (68.8)	0.050
Female	32 (40.0)	17 (53.1)	15 (31.2)	
**Hb level, g/dL**	9.7 (4.4-15.9)	9.1 (4.4-13.7)	10.0 (5.3-15.9)	0.048
**ANC level, ×10^9^/L**	2689 (646-13170)	2637 (646-7830)	2803 (1106-13170)	0.149
**PLT count, ×10^9^/L**	191.5 (53-487)	178.5 (72-487)	210.0 (53-358)	0.961
**LDH level, IU/L**	351.5 (178-1820)	377.5 (228-864)	333.5 (178-1820)	0.798
< ULN	55 (68.8)	20 (62.5)	35 (72.9)	0.325
≥ ULN	25 (31.2)	12 (37.5)	13 (27.1)	
**β2-MG level, mg/L**	3.47 (0.56-24.8)	3.60 (0.60-13.4)	3.36 (0.56-24.8)	0.321
< 5.5	60 (75.0)	25 (78.1)	35 (72.9)	0.598
≥ 5.5	20 (25.0)	7 (21.9)	13 (27.1)	
Albumin level, g/dL	3.80 (1.90-4.90)	3.35 (2.20-4.70)	3.90 (1.90-4.90)	0.016
< 3.5	50 (62.5)	14 (43.8)	36 (75.0)	0.005
≥ 3.5	30 (37.5)	18 (56.2)	12 (25.0)	
**M-protein isotype**				
IgG	48 (60.0)	17 (53.1)	31 (64.6)	0.305
Non-IgG	32 (40.0)	15 (46.9)	17 (35.4)	
**BM plasma cell, %**				
< 60	62 (77.5)	24 (75.0)	38 (79.2)	0.662
≥ 60	18 (22.5)	8 (25.0)	10 (20.8)	
Cytogenetic risk^*^				
Others	64 (80.0)	26 (81.2)	38 (79.2)	0.819
High	16 (20.0)	6 (18.8)	10 (20.8)	
**ISS risk group**				
I or II	60 (75.0)	25 (78.1)	35 (72.9)	0.598
III	20 (25.0)	7 (21.9)	13 (27.1)	
**R-ISS risk group**				
I or II	70 (87.5)	29 (90.6)	41 (85.4)	0.732
III	10 (12.5)	3 (9.4)	7 (14.6)	
**No. of previous CTx**				
1	59 (73.8)	27 (84.4)	32 (66.7)	0.078
≥ 2	21 (26.2)	5 (15.6)	16 (33.3)	
**Status at transplantation**				
Non-CR	36 (45.0)	15 (46.9)	21 (43.8)	0.783
CR	44 (55.0)	17 (53.1)	27 (56.2)	
**Conditioning regimen**				
Bu/Cy	10 (12.5)	2 (6.2)	8 (16.7)	0.301
Mel	70 (87.5)	30 (93.8)	40 (83.3)	

ANC: absolute neutrophil count, BM: bone marrow, BU: busulfan, CMV: cytomegalovirus, CR: complete response, CTx: chemotherapy, Cy: cyclophosphamide, Hb: hemoglobin, ISS: international staging system, LDH: lactate dehydrogenase, LOD: limit of detection, Mel: melphalan, MG: macroglobulin, PLT: platelet, R-ISS: revised international staging system, ULN: upper limit of normal. * High-risk cytogenetics was defined as t(4;14), t(14;16), del(17/17p), TP53 deletion, or chromosome 1 abnormalities, including gain(1q) and del(1p).

**Table 3:** Univariable and multivariable analyses of OS in multiple myeloma

Prognostic factors	Univariable			Multivariable		
	HR	95% CI	*P*	HR	95% CI	*P*
Age, years	0.991	0.948, 1.037	0.707			
Hb level, g/dL	0.864	0.726, 1.028	0.100			
PLT count, ×10^9^/L	0.995	0.990, 1.001	0.112			
BM plasma cell, > 60%	1.121	0.414, 3.030	0.822			
M-protein type, non-IgG	1.931	0.822, 4.538	0.131	2.614	1.037, 6.587	0.042
Albumin level, g/dL	0.896	0.474, 1.692	0.735	0.550	0.249, 1.218	0.141
ß2-microglobulin level, mg/L	1.043	0.944, 1,153	0.404			
LDH, ≥ ULN	2.894	1.264, 6.623	0.012	2.483	1.046, 5.893	0.039
Cytogenetics, high risk^*^	4.213	1.709, 10.388	0.002	2.404	0.936, 6.178	0.068
R-ISS, stage III	2.459	0.897, 6.737	0.080			
CMV reactivation, ≥ LOD	0.329	0.111, 0.976	0.045	0.213	0.062, 0.739	0.015
Failure to achieve CR	1.176	0.519, 2.664	0.695			

BM: bone marrow, CMV: cytomegalovirus, CR: complete response, Hb: hemoglobin, LDH: lactate dehydrogenase, LOD: limit of detection, OS: overall survival, PLT: platelet, R-ISS: revised international staging system, ULN: upper limit of normal. ^*^ High-risk cytogenetics was defined as t(4;14), t(14;16), del(17/17p), *TP53* deletion, or chromosome 1 abnormalities, including gain(1q) and del(1p).

**Table 4:** Univariable and multivariable analyses for OS in relation to late CMV reactivation (overall patients)

Prognostic factors	Univariable			Multivariable		
	HR	95% CI	*P*	HR	95% CI	*P*
Age, years	1.021	0.987, 1.056	0.230			
Hb level, g/dL	0.899	0.786, 1.027	0.117			
PLT count, ×10^9^/L	0.998	0.994, 1.002	0.293			
LDH level, ≥ ULN	1.841	0.920, 3.684	0.085	2.251	1.095, 4.627	0.027
Diagnosis of lymphoma	0.532	0.253, 1.118	0.096	0.389	0.181, 0.837	0.016
Failure to achieve CR	1.633	0.822, 3.242	0.161			
Previous CTx lines, ≥ 2	1.365	0.677, 2.753	0.385			
Late CMV reactivation, ≥ LOD	2.665	0.933, 7.615	0.067	2.964	1.014, 8.667	0.047

CMV: cytomegalovirus, CTx: chemotherapy, CR: complete response, Hb: hemoglobin, LDH: lactate dehydrogenase, LOD: limit of detection, OS: overall survival, PLT: platelet, ULN: upper limit of normal.

**Table 5:** Multivariable logistic regression analysis of risk factors for late CMV reactivation

Prognostic factors	Coefficient		OR	95% CI	*P*	Points
	Estimate	SE				
T-cell lymphoma	2.140	0.981	8.499	1.242, 58.151	0.029	1
Previous CTx lines, ≥ 2	2.197	0.995	8.995	1.280, 63.216	0.027	1
Failure to achieve CR	1.963	0.908	7.124	1.202, 42.210	0.031	1
Early CMV reactivation	2.554	0.952	12.853	1.990, 82.996	0.007	1.5

CMV: cytomegalovirus, CR: complete response, CTx: chemotherapy, SE: standard error.
